# Lipid Composition of Cell Membranes and Its Relevance in Type 2 Diabetes Mellitus

**DOI:** 10.2174/157339912802083531

**Published:** 2012-09

**Authors:** Rob N.M. Weijers

**Affiliations:** Teaching Hospital, Onze Lieve Vrouwe Gasthuis, The Netherlands

**Keywords:** Cell Membranes, Erythrocyte Deformability, Glucose Effectiveness, Glucose Transporter, Insulin Sensitivity, Phospholipids, Type 2 Diabetes Mellitus, Unsaturated Fatty Acid.

## Abstract

Identifying the causative relationship between the fatty acid composition of cell membranes and type 2 diabetes mellitus fundamentally contributes to the understanding of the basic pathophysiological mechanisms of the disease. Important outcomes of the reviewed studies appear to support the hypotheses that the flexibility of a membrane determined by* the ratio of (poly)unsaturated to saturated fatty acyl chains of its phospholipids* influences the effectiveness of glucose transport by insulin-independent glucose transporters (GLUTs) and the insulin-dependent GLUT4, and from the prediabetic stage on a shift from unsaturated towards saturated fatty acyl chains of membrane phospholipids directly induces a decrease in glucose effectiveness and insulin sensitivity. In addition, it has become evident that a concomitant increase in stiffness of both plasma and erythrocyte membranes may decrease the microcirculatory flow, leading ultimately to tissue hypoxia, insufficient tissue nutrition, and diabetes-specific microvascular pathology. As to the etiology of type 2 diabetes mellitus, a revised hypothesis that attempts to accommodate the reviewed findings is presented.

## INTRODUCTION

This report continues our analyses of the biochemical factors playing an important role in the pathogenesis of gestational diabetes mellitus (GDM) and type 2 diabetes mellitus, that is, a relationship among insulin sensitivity, glucose effectiveness, and membrane flexibility [1]. The minimal model of glucose disappearance from a frequently sampled intravenous glucose tolerance test will be the key to assessing insulin sensitivity and glucose effectiveness *in vivo*, in physiological, pathophysiological, and epidemiological studies [2]. Assessment of these parameters has revealed that type 2 diabetes mellitus and its prediabetic phase are characterised by a decrease in both glucose effectiveness and insulin sensitivity [2]. In this context, the results of a number of studies are consistent with the notion that the fatty acid composition of skeletal muscle phospholipids influences insulin sensitivity [3–5]. As mentioned by Borkman *et al.*, other authors have reached alternative conclusions, suggesting that insulin sensitivity may not be directly related to fatty acid composition at all: rather, the composition may simply be a marker for the effect of some unidentified third factor that modulates insulin sensitivity [3]. So far, no data are available describing a relationship between glucose effectiveness and the fatty acid composition of cell membrane phospholipids. The last decade has seen tremendous advances in our understanding of membranes from biological sources through exploitation of their phospholipids by volumetric measurement [6], diffuse x-ray scattering from oriented stacks of bilayers, modern liquid crystallography of lipid bilayers [7], molecular dynamics calculations [8], and quantum mechanical and empirical force field-based calculations [9]. The goal of this paper is to provide a basis for understanding the intimate association of type 2 diabetes mellitus characterised by insulin sensitivity and glucose effectiveness, and the mechanical effects of a shift from unsaturated towards saturated fatty acids in membrane phospholipids on these entities.

## 1 BERGMAN CONCEPT

The minimal model approach analyses the relationship between the pattern of insulin response and the rate of glucose decline to infer the sensitivity of tissues to insulin (S_I_) [2]. Additionally, the model can measure a relevant factor, less well recognised, and called glucose effectiveness (S_G_). This term describes the ability of glucose per se, independent of changes in insulin concentration, to stimulate its own uptake by a mass action effect and to suppress its own release [10]. With this model, S_I_ and S_G_ are measured by computer analysis of the frequently sampled intravenous glucose tolerance test involving intravenous injection of glucose followed by tolbutamide or insulin and frequent blood sampling. Insulin sensitivity and glucose effectiveness are expressed in compatible units, *i.e*., S_I_(×10^-4^·min^-1^·mU^-1^·L) and S_G_(min^-1^), respectively. Although the absolute values of the parameters S_I_ and S_G_ are still being debated, values of S_G_ average 0.021 min^-1 ^in humans and mean S_I _in normal volunteers have remained at approximately 5.1×10^-4^·min^-1^·mU^-1^·L in several studies of human volunteers [2]. In the average volunteer, whose plasma insulin increases by 70 mU/L after a carbohydrate load, the restoration rate is given as glucose×(0.021+5.1×10^-4^×70) = glucose×(0.021+0.036). In this average individual, most of the glucose restoration is attributable to insulin [0.036/(0.021+0.036)=63%], whereas the remaining 37% is attributable to glucose effectiveness, independent of insulin. In the basal state, healthy individuals have insulin oscillations with a regular 14-min periodicity of amplitude of 1.8 mU/L [11]. Thus, most of the restoration of glucose results from the ability of glucose to stimulate its own uptake: glucose×[0.021/(0.021+0.00092)=0.96%], whereas the remaining 4% is attributable to insulin sensitivity. These data strongly suggest that glucose effectiveness is relevant to the physiological understanding of all mechanisms for lowering a high blood glucose concentration that operates independently of a plasma insulin increase.

## 2 GLUCOSE EFFECTIVENESS AND INSULIN SENSITIVITY DURING THE PREDIABETIC AND DIABETIC STAGES OF TYPE 2 DIABETES MELLITUS

Studies using computer modelling of glucose and insulin kinetics after intravenous glucose challenge have demonstrated that patients with type 2 diabetes have significantly lower values of both insulin-independent glucose removal rate (S_G_) and insulin sensitivity (S_I_) than those who remain normoglycaemic (Table **1**) [2, 12–14]. In addition, glucose-induced stimulation of its own uptake is abnormal in type 2 diabetes, but glucose-induced suppression of endogenous glucose production and output is not [15].

A prospective study on the development of type 2 diabetes in normoglycaemic offspring of couples who both had type 2 diabetes showed defects in glucose disposal and insulin sensitivity, that is, more than 10 years before the development of diabetes, participants who developed the disease had lower values compared to controls of both S_I_ ([3.2 ± 2.4 *vs.* 8.1 ± 6.7] ×10^-3^·min^-1^·pmol^-1^ insulin; *P *< 0.0001) and S_G _([1.6 ± 0.9 *vs.* 2.3 ± 1.2] × 10^-2^·min^-1^; *P *< 0.0001) [14]. Those findings suggest that the traits that characterise the prediabetic stage may be genetically determined.

An unanswered question arises: Is there a fundamental relationship between diminished glucose effectiveness and diminished insulin sensitivity in people in the prediabetic phase and people with type 2 diabetes, and if so, what is that relationship? This review develops a model that combines glucose effectiveness and insulin sensitivity into one fundamental biochemical principle.

## 3 GLUCOSE TRANSPORT ACROSS CELL MEMBRANES

Specific transporter proteins (Solute carrier family 2, facilitated glucose transporter members, GLUTs) are required for facilitated glucose diffusion into cells [16]. Such proteins facilitate net movement of glucose only in the thermodynamically favoured direction. Facilitated diffusion rates display saturation behaviour similar to that observed with substrate binding by enzymes. Members of the GLUT protein family that mediate facilitated glucose transport belong to a much larger superfamily of 13 functional mammalian hexose carriers. In this review, we discuss for convenience GLUT1, a widely expressed isoform that provides many cells with their basal glucose requirement; GLUT2, present on β-cells, GLUT3, which is responsible for glucose uptake into neurons; and GLUT4, which is expressed exclusively in the insulin-sensitive tissues, fat and muscle. 

GLUTs are integral membrane proteins that contain 12 membrane-spanning helices with both the amino and carboxyl termini exposed on the cytoplasmic side of the membrane. The three-dimensional structure of GLUT1 as obtained by homology modelling consists of eight helices immersed in a box formed by the remaining four helices. From the extracellular side, GLUT1 dimensions are about 36×26 Ǻ, from the cytoplasmic side, they are about 46×27 Ǻ [17]. Consequently, one molecule GLUT1 with a mean surface of about 1,100 Ǻ^2^ covers an area of about 17 molecules of a phosphatidylcholine (PC) bilayer with saturated fatty acyl chains (Table **2**).

For simplicity, we assume that classical Michaelis–Menten kinetics are valid to describe the unidirectional transport of glucose across the membrane by the high-affinity transporters GLUT1, GLUT3, and GLUT4 with Michaelis–Menten constants (K_m_s) between 1 and 5 mmol/L, and the low-affinity transporter GLUT2 with a K_m_ of approximately 25 mmol/L. Because the K_m_s of the high-affinity GLUTs are below the normal range of blood glucose concentrations, they function at rates close to maximal velocity. Dysfunction or inadequate expression of the insulin-independent GLUT1, GLUT2, and GLUT3 has not yet been described in humans [18]. Of importance, the data suggest that the transport velocity of both GLUT1 and GLUT3 is limited only by environmental conditions (temperature, and pH) and degrees of cell surface expression greatly influence the rate of glucose uptake into cells [19].

Biophysical and structural studies indicate that interactions of membrane proteins with lipid molecules are critical to their folding and stability [20, 21]. Changes in the phospholipid fatty acid composition of membranes will result in changes in the collective physicochemical properties of the bilayer, such as flexibility and fluidity. We therefore suggest that the fatty acid composition of membrane phospholipids is a cellular factor that may influence glucose transport by the insulin-independent GLUTs, that is, glucose effectiveness.

Regarding the insulin-dependent GLUT4, polymorphisms in the *SLC2A4* (*GLUT4*) gene are rare in type 2 diabetes and have the same prevalence among non-diabetic persons, suggesting they are population variants and do not play a role in the aetiology of type 2 diabetes mellitus [22,23]. Overall, levels of GLUT4 expression are normal in muscle of diabetic individuals [24], and primary defects in glucose transport all appear to be extremely rare [25].

GLUT4 differs from other glucose transporters in that about 90% of it is sequestered in intracellular vesicles in the absence of insulin. On stimulation by insulin, the intracellular stores are translocated to muscle plasma membranes, the principal site of insulin-mediated glucose disposal [26]. A cascade of events such as tethering, docking *via* highly conserved membrane-anchored proteins (SNAREs) and triggering culminates finally in membrane fusion with GLUT4 containing vesicles [27].

As described by the ‘stalk-pore’ hypothesis, membrane fusion is a localised event in which two adjacent membranes approach one another, establish a microscopic region of ‘molecular contact’, bend into sharply curved transient structures, break the transmonolayers to form a fusion pore, and eventually merge into one continuous membrane [28]. This process demands flexibility of the membrane, which is largely governed by the thermotropic state of the hydrocarbon interior, the lateral diffusion coefficient of the lipid molecules, and the spontaneous curvature of the membrane leaflets to overcome the activation energy barrier [29,30]. Consequently, the rate and extent of fusion depend on the propensity of the corresponding monolayers of membranes to bend in the required directions [31]. Furthermore, lipid–lipid van der Waals interactions are not without costs because the higher the affinity, the more energy required to dissociate such interactions. Those studies led to the hypothesis that independent of the insulin concentration the degree of skeletal muscle–membrane flexibility governed by the fatty acid composition of its phospholipids primarily determines the extent of successful fusions of GLUT4 containing vesicles with the plasma membrane. We therefore suggest that insulin sensitivity is a dependent variable of the composition of fatty acid molecules in a phospholipid bilayer of defined membrane structure.

## 4 CELL MEMBRANES

Phospholipids are the major constituents of the biological membranes, and glycerophospholipids are the major class of naturally occurring phospholipids. A variety of polar groups are esterified to the phosphoric acid moiety of the molecule. The phosphate, together with such esterified entities, is referred to as a hydrophilic headgroup. The two fatty acyl chains yield a roughly cylindrical molecule (the hydrocarbon region) that can easily pack in parallel to form extended sheets of membranes. In 1972, Singer and Nicolson proposed the fluid mosaic model for membrane structure, which suggested that membranes are dynamic structures composed as a mosaic of proteins and phospholipids in a fluid phospholipid matrix [32].

**Fig. (1):**
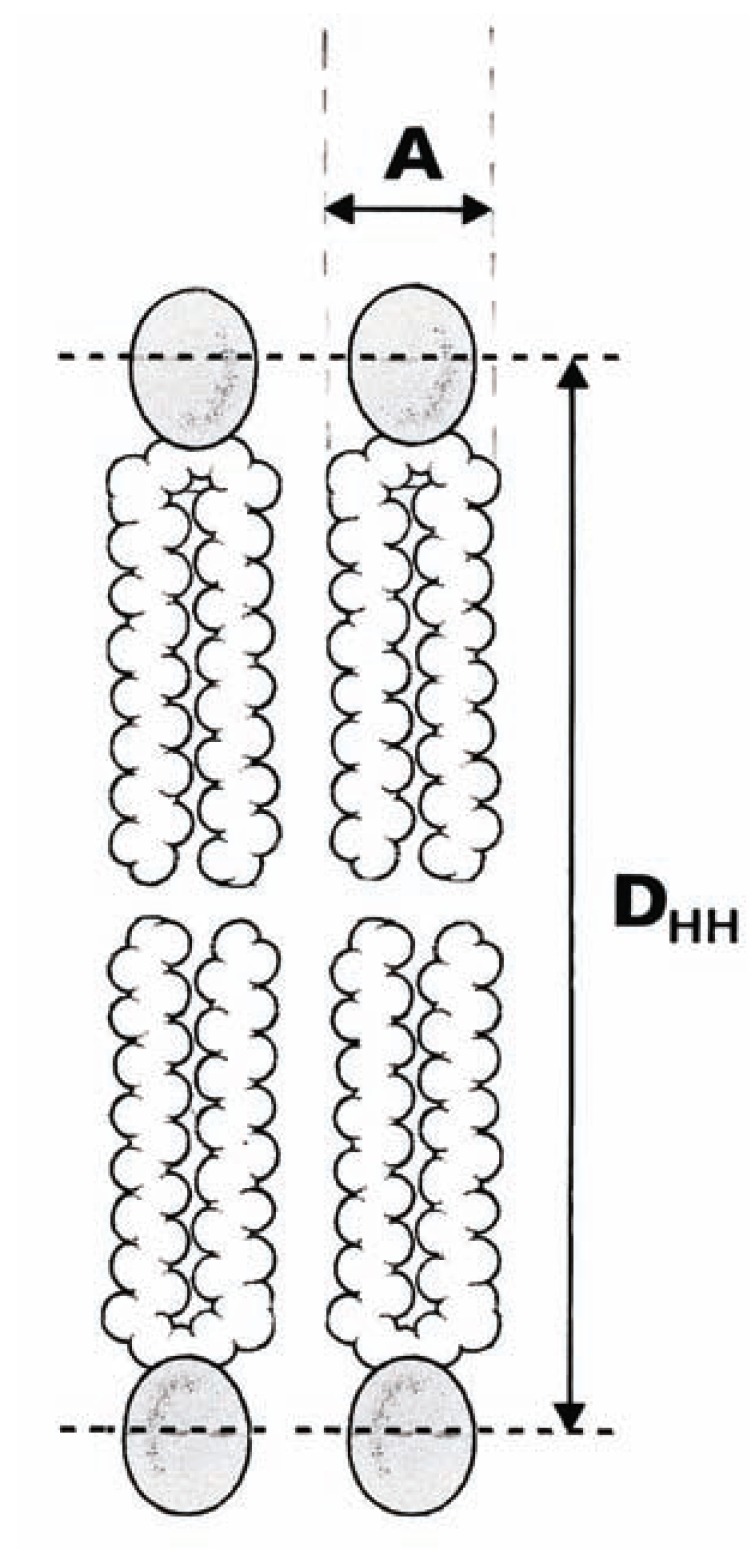
Schematic representation of part of a lipid bilayer. A represents the interfacial area A per lipid molecule, that is, the surface of the cross-section of the cylindrical part of the phospholipid molecule, and D_HH_ the head–head distance across the lipid bilayer.

The most basic structural result obtained by x-ray scattering from oriented bilayers in model membrane systems is a particularly central quantity, the area (A) per lipid molecule, that is, the surface of the cross-section of the cylindrical part of the phospholipid molecule [6,7]. The volume of the hydrocarbon chain region (V_C_) has been estimated from the difference in total volumes of the lipid molecule and the headgroup [33, 34]. Finally, values of A are obtained by assaying the half thickness of the hydrocarbon chain region (½ D_HH_) (Fig. **1**) using the electron density profiles for various samples of PC bilayers (Table **2**). We note that unsaturation compared to saturation clearly leads to a larger value for A (in the case of dioleoyl-phosphatidylcholine (DOPC) [di(C18:1)PC] and egg phosphatidylcholine (EPC) *versus* dipalmitoyl-phosphatidylcholine (DPPC) [di(C16:0)PC], dimyristoyl-phosphatidylcholine (DMPC) [di(C14:0)PC], and dilauroyl-phosphatidylcholine (DLPC) [di(C12:0)PC].

A shift from unsaturation towards saturation also results in changes in the collective physical properties of the headgroup regions of the bilayer. For example, at full hydration, a bilayer of DOPC [di(C18:1)PC] with A=72.5 Ǻ^2^ takes up about 11 molecules of water per headgroup molecule, whereas a bilayer of DMPC [di(C14:0)PC] with A=60.6 Ǻ^2^ takes up only about 7 molecules of water per headgroup molecule (Table **2**). In addition, the electrostatic interactions between charged PC headgroups of unsaturated phospholipids will be less than that between PC headgroups of saturated phospholipids because the strength of electrostatic interactions between two PC headgroups begins to fall at 1/r.

A closely related phenomenon is the effect of the degree of bilayer unsaturation on its surface tension. Analysis of an extensive set of molecular dynamics simulations on explicit lipid bilayers has demonstrated that surface tensions may be significantly lower in bilayers with unsaturated compared to saturated fatty acyl chains: for example, the surface tensions of DOPC [di(C18:1)PC] and 1-palmitoyl-2-oleoyl-PC (POPC) [(C16:0,C18:1)PC] bilayers are 0.012 and 0.019 N/m, respectively, while those of DMPC [di(C14:0)PC] and DPPC [di(C16:0)PC] bilayers are 0.047 and 0.027 N/m, respectively [8]. This concept can be understood in terms of the forces present within a lipid bilayer. At about the position of the glycerol backbone region, just below the lipid headgroups, an attractive force Fγ arises from the unfavourable contact of the hydrocarbon chains with water. Tight packing in this region ensures a minimum exposure of the hydrocarbon interior of the membrane to water, leading to a positive membrane tension, tending to contract the bilayer**.** The presence of *cis*-unsaturated fatty acyl chains in the component lipids decreases the tight packing and consequently decreases the positive membrane tension [20].

## 5 STRUCTURE OF SATURATED *VERSUS* UNSATURATED FATTY ACIDS

Saturated fatty acids have essentially linear alkyl chains. Double bonds in unsaturated fatty acids are almost always in the *cis* configuration, which produces a bend in the fatty acid chain. Molecules like palmitoleic acid (C16:1) and oleic acid (C18:1) are bent at the *cis *double bond, and the two chain parts form an angle of 113° [35-38]. This association is consistent with the crystal structures of various long-chain unsaturated fatty acids showing a “kink” at the *cis* double bond, which creates a more open structure between the fatty acid chains [35, 39].

**Fig. (2):**
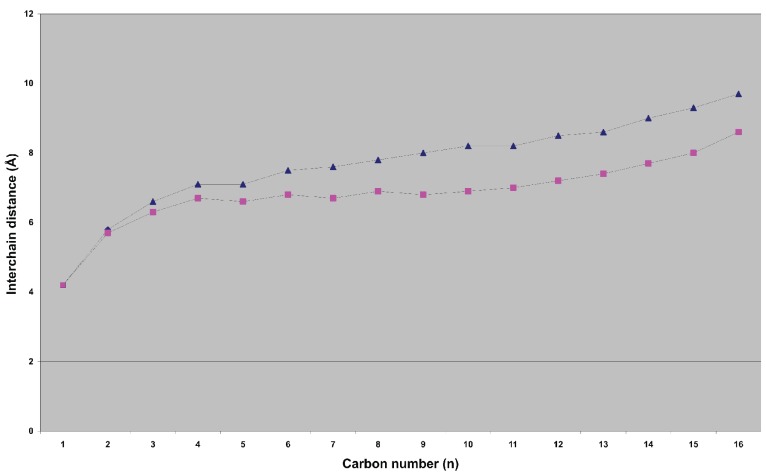
Average interchain distances for the simulation of PEPC (■) and POPC (▲). PEPC: 1-palmitoyl-2-elaidoyl-phosphatidylcholine; POPC: 1-palmitoyl-2-oleoyl-phosphatidylcholine.

Using Langevin dynamics, computer simulations of phospholipids in a membrane environment have been carried out to derive their average fatty acid interchain distances, such as simulations of 1-palmitoyl-2-elaidoyl-phosphatidylcholine [(C16:0,* trans-*C18:1)PC] (PEPC) and 1-palmitoyl-2-oleoyl-phosphatidylcholine [(C16:0,* cis-*C18:1)PC] (POPC) [40]. In POPC, the two chains begin to separate more starting from the carbons in the fifth position and are almost an angstrom further apart at the ends of the chains than those of PEPC (Fig. **2**). Numerous inter-atomic details have made it fairly clear that PEPC [(C16:0,C18:1)PC] closely resembles DPPC [di(C16:0)PC] in most of its attributes, implying that the presence of the *trans* double bond of elaidic acid [41] influences the behaviour of the chain very little, as the torsions in a saturated chain prefer a mostly *trans* geometry [42]. Therefore, the calculated average interchain distances of PEPC [(C16:0,C18:1)PC] and POPC [(C16:0,C18:1)PC] are transferable to the extra space created in membranes by the acyl chain of oleic acid as compared with a saturated acyl chain. ^2^H nuclear magnetic resonance measurements have revealed that *cis *double bonds may cause the fatty acyl chains to occupy a slightly wedge-shaped space, leading to looser packing at the lipid–water interface [42]. Furthermore, molecular dynamics simulation and quantum mechanical calculations of a 1-stearoyl-2-docosahexaenoyl-phospatidylcholine [(C18:0,C22:6)PC] lipid bilayer model have indicated an unusually high degree of conformational flexibility of the polyunsaturated hydrocarbon chain in phospholipid membranes (docosahexaenoic acid is a fatty acid present in high concentrations in cell membranes from neural tissues such as the brain and retina) [9].

## 6 LONDON-VAN DER WAALS INTERACTION ENERGY

In aqueous solution, the two layers of phospholipid molecules self-assemble so that their hydrophilic heads form the surfaces at the exterior and the interior of the membrane, and the large hydrocarbon hydrophobic tails face each other inwardly. The polar headgroups are hydrated in water, while the buried hydrocarbon tails interact with each other through London-van der Waals forces [43, 44]. The Lennard-Jones potential is a mathematically straightforward model that describes the interaction between a pair of neutral atoms or molecules. The interaction energy U between two carbon atoms is given by

Equation (1) $$U={B/r}^{12}-{A/r}^{6}$$

where r is the distance between the centres of two carbon atoms. The values for the parameters B=11.5×10^-6^ kJ·nm^12^/mol and A=5.96×10^-3^ kJ·nm^6^/mol for the interaction between two carbon atoms are taken from M. Levitt [45]. The r^-12^ term describes Pauli repulsion at short ranges resulting from overlapping of electron orbitals, and the r^-6^ term describes the attraction at longer interatomic distances (van der Waals force). 

To estimate the interaction energy of the saturated phospholipid DMPC [di(C14:0)PC], we used its area per lipid molecule, A=60.6 Ǻ^2^ and calculated an interchain carbon–carbon distance of 4.39 Ǻ. Using Equation (1), the interaction energy U is U_DMPC_=–0.61 kJ/mol. Regarding the unsaturated phospholipid DOPC [di(C18:1)PC] with an area per lipid molecule of 72.5 Ǻ^2^ and an interchain carbon–carbon distance of 4.80 Ǻ, the interaction energy U is U_DOPC_=–0.41 kJ/mol (Table **2**). Thus, replacement of unsaturated DOPC with saturated DMPC results in a 32.8% increase in interaction energy per a pair of fatty acyl carbon atoms. These data show that a larger area A per lipid molecule, resulting from unsaturation, leads to decreased membrane rigidity and increased membrane flexibility. For similar reasons, trioleoyl-glycerol is a liquid and tristearoyl-glycerol is a solid melting at 71ºC.

## 7 ERYTHROCYTE DEFORMABILITY

One of the hemorheological parameters altered in diabetes mellitus type 2 is the deformability of erythrocytes, which physiologically depends on the surface–volume ratio, internal viscosity and dynamic properties of the erythrocyte membrane [46-48]. Deformability is a relatively general term that describes the ability of a body (erythrocytes in this case) to change its shape in response to a deforming force. Erythrocyte deformability of patients with type 2 diabetes has been found to be significantly reduced compared with healthy participants [49]. Furthermore, a study of 64 patients with longstanding type 2 diabetes and 61 matched non-diabetic participants demonstrated decreased erythrocyte deformability in the 14 diabetic patients with the most extensive micro-angiopathy as compared to the 22 diabetic patients with slight or no complications or to non-diabetic controls [50]. 

Min *et al.* studied the fatty acid composition of red cell membrane phospholipids in women with GDM [51]. Appreciable reductions in polyunsaturated arachidonic acid [C20:4] and docosahexaenoic acid [C22:6] were correlated with a substantial increase in saturated fatty acids in erythrocyte membrane PC and phosphatidyl-ethanolamine (PE) in the women with GDM compared with controls. From their data, we calculated the unsaturation index for membrane flexibility, *i.e.*, the number of *cis* fatty acyl molecules per total number of fatty acyl molecules in a phospholipid bilayer of defined membrane structure. GDM women showed a markedly lower PC and PE unsaturation index as compared with controls, (PC: 0.49 ± 0.09 *vs*. 0.54 ± 0.07, *P *< 0.01; PE: 0.67 ± 0.13 *vs.* 0.70 ± 0.09, *P *< 0.05, respectively) [1].

Because the progressive metabolic derangement of glucose tolerance during GDM mimics the pathogenesis of type 2 diabetes, these data suggest a relationship between reduced glucose effectiveness, reduced insulin sensitivity and reduced erythrocyte deformability accompanying the shift from unsaturated to saturated fatty acids in erythrocyte membrane phospholipids, features already present during the prediabetic stage. 

## DISCUSSION

A bilayer membrane will adopt a state in which the attractive interactions between the hydrocarbon chains and the repulsive interactions between the headgroups balance each other. The strength of the interactions depends on the interaction energy between pairs of hydrocarbon atoms of the phospholipid fatty acyl chains involved, and is given by the Lennard-Jones equation (Equation 1). Its potential curve shows a minimum that defines the distance known as the van der Waals contact distance, which is the interatomic distance that results if only van der Waals forces hold two atoms together. With regard to carbon atoms, this distance is 3.96 Ǻ at a minimum interaction energy of –0.77 kJ/mol. At a distance of 4.96 Ǻ the interaction energy is –0.35 kJ/mol, a decline of about 55%. Due to the term r^-6 ^(Equation 1), a small change of the distance between two hydrocarbon acyl chains in a membrane phospholipid strongly influences their van der Waals interaction energy.

Because of the presence of one or more *cis* double bounds in the hydrocarbon acyl chain of a membrane phospholipid, the area A of this lipid molecule is larger compared to a phospholipid without double bounds in its hydrocarbon acyl chain(s). An increased area A creates ‘extra space’ between the two hydrocarbon acyl chains, which in turn results in reduced interaction energy between the interchain carbon–carbon atoms, and makes the membrane more permeable to water [42]. Conversely, saturated hydrocarbon fatty acyl chains can pack closely together under certain conditions to form ordered and rigid arrays. It seems likely that the key to understanding unsaturation in phospholipid membranes lies in the membrane lateral organisation and the interfacial properties such as lipid packing, molecular cross-sectional area, the tendency for curvature, membrane permeability, and viscoelasticity [52]. Based on these observations, we suggest that a shift from unsaturated towards saturated fatty acids in phospholipid membranes counteracts both the pore formation of a muscle plasma membrane fused with a docked intracellular GLUT4 containing vesicle, and the machinery responsible for insulin-independent GLUT insertion into a plasma membrane, particularly because the GLUT interfacial area exceeds by about 17-fold the area A of membrane phospholipids. In this way, the unsaturation index unifies insulin sensitivity and glucose effectiveness into one model system.

A generalized representation of the cross-sectional phospholipid hydrocarbon chain packing of a cell membrane is a regular hexagonal array [53, 54]. Like all naturally occurring systems proceed towards equilibrium, that is, to a state of minimum energy, the hexagonal ordered state is a low-energy state. The energy requirement of the insertion of a phospholipid molecule into a biological membrane exhibits a compositional dependence on area A, in that it predicts a lower insertion energy of a phospholipid with a smaller area A compared with a phospholipid with a larger area A. Thus, especially in individuals with a defect in skeletal muscle ATP production the insertion of saturated phospholipids as compared with unsaturated phospholipids is in favour during the prediabetic and diabetic phase.

**Fig. (3):**
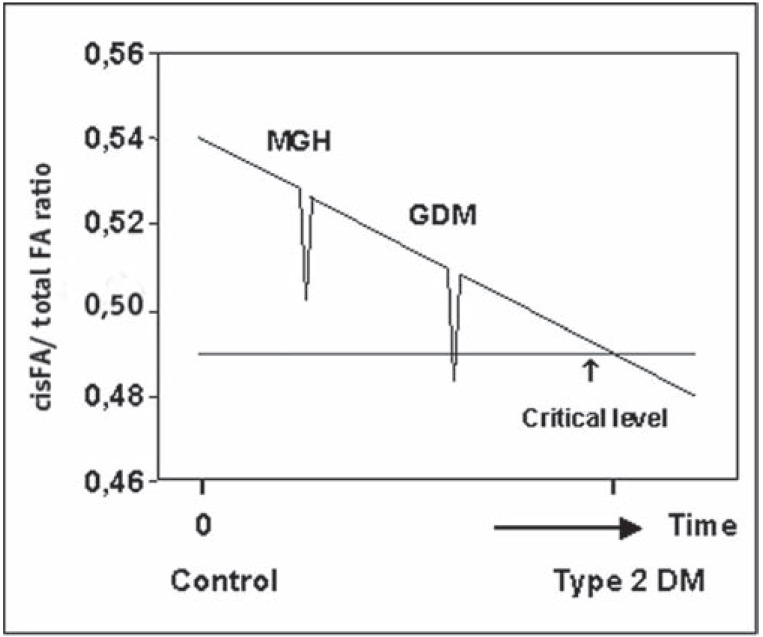
Hypothetical relationship between the ratio of *cis* fatty acid (*cis* FA) to total FA and the duration of the prediabetic stage. The *cis* FA/total FA ratio is that of PC and PE fatty acids in the erythrocyte membrane. At time 0, the value of the ratio represents the value for pregnant women with normal glucose tolerance and no inherited defect in mitochondrial oxidative phosphorylation. Reduction of the ratio below a critical value is postulated to result in overt type 2 diabetes mellitus. The distribution of the y axis values is based on previously published data [1]. The ratio is assumed to decrease in pregnant women because of increased plasma saturated free fatty acid concentrations, resulting in mild gestational hyperglycemia, GDM, or type 2 diabetes mellitus.

Support for our model comes from a number of studies. First, Cline *et al.* demonstrated that transmembrane glucose transport is the rate-controlling step in insulin-stimulated muscle glycogen synthesis in patients with type 2 diabetes mellitus [55]. They also found no difference in the interstitial-fluid insulin concentrations during the steady-state of hyperinsulinemic clamp studies in the patients with type 2 diabetes as compared with normal subjects, suggesting that the delivery of insulin is not responsible for the approximately 80 percent lower rate of glucose infusion in the patients with type 2 diabetes compared with that of normal subjects. An important outcome of the work by Cline *et al.* concerns the insulin-independent reduction in glucose metabolism and glycogen synthesis and most probable a lipid compositional change of the cell membrane. Second, Garvet *et al.* subfractionated on discontinuous sucrose density gradients to equilibrium muscle membranes obtained under basal conditions and found a significant enrichment of GLUT4 in *denser* membrane fractions of type 2 diabetics compared with insulin-sensitive controls without any statistically significant differences in the recovery of membrane markers in any of the subfractions [56]. Additionally, no effects of insulin stimulation on GLUT4 localization were observed. An explanation may be a shift from unsaturated towards saturated fatty acyl chains of membrane phospholipids, which creates an increase in the van der Waals force and a more tight packing of phospholipids resulting in an increase in their characteristic densities (ρ = m/V), a physical variable on which the separation strategy of gradient centrifugation relies. Third, we looked at the relationship between GDM and type 2 diabetes [1]. One of the characteristics of the second half of pregnancy is an appreciable increase in the maternal plasma concentration of free fatty acids, which is thought to be the main cause of a decrease in insulin sensitivity and in the unsaturation index [57-59]. We suggest that a reduction in the unsaturation index in pregnant women with GDM is the summation of two indexes: the typical temporal decrease in index seen in late pregnancy, and a more chronic decrease seen in the prediabetic stage of type 2 diabetes mellitus (Fig. **3**). Depending on the phase of the latter, superposition of both indices may result in an index value characteristic of *mild* gestational diabetes (MGD) or GDM [1]. Fourth, well documented are a reduced erythrocyte deformability in patients with type 2 diabetes mellitus and a longstanding prediabetic shift from unsaturated to saturated fatty acids in erythrocyte membrane phospholipids [47, 50]. In contrast to the prevailing view that increased glycation creates reduced erythrocyte deformability [47], the main cause may be a more tight packing of saturated fatty acyl chains of phospholipids. In fact, the driving force of membrane flexibility is formed by the biomolecule with the lowest stabilization energy. Because the electrostatic interactions within a folded protein structure contribute about 20 kJ/mol of stabilization energy and the van der Waals interactions between fatty acyl chains individually contribute 0.4 – 4.0 kJ/mol of stabilization energy, the latter is essential to the maintenance of the erythrocyte deformability [16]. It is interesting to note that the erythrocyte membrane is compositionally very similar to the vascular endothelium [51]. Thus, it is likely that if the erythrocyte membrane is selectively affected in type 2 diabetes mellitus, the endothelium could also be affected with the expected consequences of vascular dysfunction [60, 61]. In capillaries in which the size of erythrocytes is of the same order of magnitude as the lumen, *i.e.,* approximately 8 μm, deformability is an important determinant of blood flow. The increased stiffness of both erythrocyte and plasma membranes may decrease the microcirculatory flow, leading ultimately to chronic tissue hypoxia, insufficient tissue nutrition**,** and diabetes-specific microvascular pathology in the retina, renal glomerulus and peripheral nerve. The primary cause of microangiopathy may thus be due by two processes both mediated by a shift from unsaturated towards saturated fatty acids in membrane phospholipids. The first process results in both reduced glucose effectiveness and insulin sensitivity which diminishes transmembrane transport of glucose, the starting molecule for ATP production in the cell. The second process is the sequence of events ending in increased stiffness both of erythrocytes and plasma membranes, which leads to diminished oxygen transport and stagnating of oxidative phosphorylation, the final phase of ATP production in the cell [16]. Fifth, in our proposed hypothesis a reduction in free fatty acids implicates an increase in unsaturation index, membrane flexibility, glucose effectiveness, and insulin sensitivity. This sequence of events is supported by the outcomes of observational studies and clinical trials of diet, exercise, or both in persons at high risk for diabetes mellitus demonstrating that lifestyle changes reduce the incidence of the disease in persons at high risk [62-64]. Finally, Borkman *et al.* has described in a group of 13 normal men a positive correlation between the concentration of long-chain polyunsaturated fatty acids within skeletal muscle phospholipids and the index of insulin sensitivity [3]. Because phospholipids are confined to membranes and there is evidence of a continuous and rapid transfer of phospholipids between membranes [65], these data may indicate that a shift from saturated towards unsaturated phospholipids in the skeletal muscle membrane facilitates the fusion process of plasma membrane with intracellular GLUT4 sequestered vesicles. Because of less rigid plasma membranes, more successful vascular fusions would occur at a fixed concentration of insulin, ending in a higher number of GLUT4 transporters per area of plasma membrane: in other words, ending in an increased insulin sensitivity.

An important issue of this study concerns the reliability of the presented physicochemical data. Many prominent biophysicists have published *experimental methods* for obtaining lipid bilayer structures and experimental data characterizing those bilayer structures including a particularly central quantity, that is, the area A per lipid molecule [6, 7, 20]. On the other hand, they used *computer simulations* to study the motions of single phospholipid molecules [9, 28, 40, 66]. The predictions of those computer simulations are in qualitative agreement with the results of experimental methods. Good examples are: (1) calculations using statistical thermodynamic methodology performed for DPPC [di(C16:0)PC] and DOPC [di(C18:1)PC] predict for the latter an increase in A of 10.4 Ǻ^2 ^[66], whereas Nagle *et al. *[7] reported an increase of 8.5 Ǻ^2 ^(from 64 to 72.5 Ǻ^2^) based on experimental bilayer structures (Table **2**); (2) calculations using Langevin dynamics predicted that in POPC [(C16:0,C18:1)PC] the two chains begin to separate more starting from the carbons in the fifth position and are nearly one angstrom further apart at the ends of the chains [40], whereas Nagle *et al.* [7] comparing DPPC [di(C16:0)PC and DOPC[di(C18:1)PC] reported a mean increase of 0.8 Ǻ (from 8.8 to 9.6 Ǻ). The results of both methodologies underscore that the use of mathematics to understand the reality has become an integral part of science.

Our findings in this report are contradictory to some suggestions regarding possible risk factors for type 2 diabetes that modulate insulin sensitivity. First, insulin deficiency indicates that insulin has a permissive effect on fatty acid desaturase activity [67, 68]. However, the biosynthesis of polyunsaturated fatty acids of the ω6 series involves the sequence linoleic acid 18:2 ω6 → linolenic acid 18:3 ω6 → dihomogamma linolenic acid 20:3 ω6 → arachidonic acid 20:4 ω6. Because the so far known predicted equilibrium average molecular areas of these polyunsaturated lipida are of the same order of magnitude [66], their contribution to the flexibility of the bilayer membrane is largely identical. Second**,** insulin resistance (I prefer the expression; reduced insulin sensitivity) may result from hyperinsulinemia [69, 70]. The cause is a tighter packing of membrane phospholipids due to an increase in saturated phospholipid fatty acyl chains and the effect is an increased plasma insulin concentration (see for detailed information the second paragraph of the section “Revised hypothetical steps in the development of type 2 diabetes mellitus”. Last, abnormalities in GLUT4 translocation in muscle appear to result from defects in intracellular signalling [26]. Based on the data of Garvey *et al.* [56], we argued that a more conclusive explanation is a shift from unsaturated towards saturated fatty acyl chains of membrane phospholipids, which creates a more tight packing of phospholipids and consequently a decrease in the capacity for GLUT4 glucose transport.

An obvious limitation of our study is one that is largely inherent to all theoretical models in biochemistry in general, which never allow for complete certainty [71]. For example, to what extent calculations based on artificial models do properly describe intermolecular interactions, especially the London-van der Waals forces? Recently, Sun *et al.* presented atomic force microscopy measurement data of intermolecular interactions between two CO molecules [72] indicating the correctness of the Lennard-Jones potential as described by London in 1937 [73]. Also, structural information about lipid bilayers, widely used as basic information to help model biomembrane structure and their functions, is obtained from model membranes simulating the mechanism of vesicle fusion [28], and the insertion and assembly of membrane proteins [74]. The simulation results of those artificial models are in good agreement with experimental data, suggesting a high degree of reliability of our picture of the lipid bilayer nature. Harland *et al.* recently found that phospholipid bilayer membranes are not simply viscous but rather exhibit viscoelasticity, which must be integrated into our still-developing understanding of two-dimensional fluids [52]. In the future, more discoveries will help to identify all phenomena that may contribute to a full understanding of what lipid membranes are precisely. Another limitation is the small sample size of participants involved in studying the fatty acid pattern of membrane phospholipids in controls and women who developed GDM, which somewhat limits the conclusions from this part of the argument for the whole population with type 2 diabetes mellitus [51]. However, the main conclusions, specifically that concerning the correlation between insulin sensitivity with the ratio of (poly)unsaturated fatty acids to saturated fatty acids in membrane phospholipids, are consistent with the notion that in healthy individuals the level of phospholipid long-chain polyunsaturated fatty acids is positively correlated with insulin sensitivity [3]. Further, they are consistent with the demonstration in isolated cells that direct alterations in the fatty acid composition of membranes induce changes in insulin responsiveness [4, 5].

## REVISED HYPOTHETICAL STEPS IN THE DEVELOPMENT OF TYPE 2 DIABETES MELLITUS

As to the aetiology of type 2 diabetes mellitus, an improved hypothesis that attempts to accommodate the discussed data is presented in Fig. (**4**) [75]. Reductions in basal rates of mitochondrial ATP synthesis in skeletal muscle due to decreased mitochondrial activity or a relative short supply of ATP in maternal circulation during pregnancy because of foetal growth are early events [76-78] and serve to drive hepatic lipogenesis. This leads to a gradual elevation of plasma free fatty acids [58, 59, 79-81] to yield more energy (in the form of ATP), which develops a shift from unsaturated towards saturated fatty acyl chains of membrane phopholipids and a harmful increase in membrane stiffness which results in a reduction in both successful insertions of insulin-independent GLUTs into plasma membrane and fusions of insulin-dependent GLUT4 containing vesicles with plasma membrane. The net effect would be a decreased flux of glucose into cells which causes a further stimulus to hepatic fatty acid production. On account of the reduction of GLUTs, a concomitant elevated plasma glucose concentration stimulates the pancreas to oversecretion of insulin and amylin [82]. The progress of these events set up a vicious cycle. The plasma glucose and insulin concentrations increase and are positively related up to a plasma glucose concentration of about 10 mmol/L. Thereafter, β-cell failure occurs, and glucose intolerance gives way to frank type 2 diabetes mellitus.

Focused on the role how the phopholipid changes in the plasma membrane composition alter the insulin secretion we recapitulate: As described by the ‘stalk-pore’ hypothesis, membrane fusion of a GLUT4 containing vesicle with a cell membrane is a localized event in which the two adjacent membranes approach, establish a microscopic region of ‘molecular contact’, bend into sharply curved transient structures, break the transmonolayers to form a fusion pore, and eventually merge into one continuous membrane [28]. (Studying Fig. (**4**) of reference 23 depicting targeting and fusion of vesicles with the target organelle will be helpful for the understanding of the events). This process demands flexibility of the cell membrane, which is largely governed by the ratio of unsaturated and saturated membrane phospholipids. A shift from unsaturated towards saturated fatty acids in phospholipid membranes increases the van der Waals forces between the hydrocarbon chains, reduces the membrane flexibility, and counteracts the fusion resulting in a reduction of successful fusions and a decrease in the capacity for GLUT4 glucose transport. In normal individuals and individuals with impaired glucose tolerance the increasing plasma glucose concentration is positively related to the insulin concentration to a mean plasma glucose concentration of about 10 mmol/L [83].

In conclusion, this review shows that a shift from unsaturated towards saturated phospholipid fatty acyl chains plays a central, if not primary, role in causing more rigid arrays of phospholipid molecules in plasma membranes, which may damage both the machinery responsible for insulin-independent GLUT insertion into plasma membrane and the fusion of muscle plasma membrane with insulin-dependent docked GLUT4 containing vesicles. As a new therapeutic option, upcoming studies need to be designed to prevent an increase in saturated fatty acyl chains of membrane phospholipids, a characteristic of type 2 diabetes mellitus and its prediabetic phase, and an essential prerequisite for slowing down the onset of the disease and the development of diabetic microangiopathy.

## Figures and Tables

**Fig. (4):**
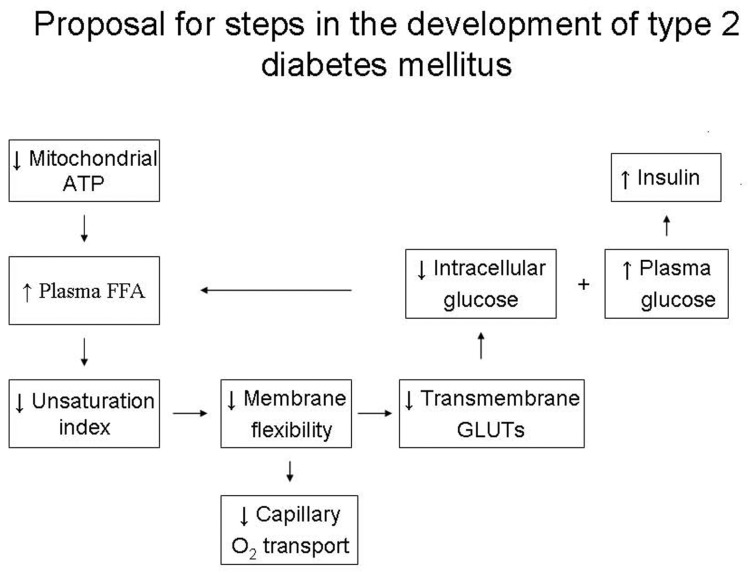
Revised hypothetical steps in the development of type 2 diabetes mellitus.

**Table 1.** Relation Between Insulin Sensitivity (S_I_)* and Glucose Effectiveness (S_G_)

	Control Subjects	Type 2 Diabetics	*P*	Ref.
S_G_(min^-1^)	0.023 ± 0.002			[2]
	0.016 ± 0.001	0.010 ± 0.001	<0.01	[12]
	0.020 ± 0.002	0.013 ± 0.001	<0.05	[13]
	0.023 ± 0.012	0.016 ± 0.009	<0.0001	[14†]
S_I_(×10^-4^·min^-1^·mU^-1^·L)	5.61 ± 0.51			[2]
	11.8 ± 2.6	6.7 ± 0.8	<0.05	[13]
	13.45 ± 11.12	5.31 ± 3.98	<0.0001	[14†]

* We used the conversion factor: 1 mU/L = 6.00 pmol/L;

† more than 10 years before the development of diabetes

**Table 2.** Experimental Data of Fully Hydrated Fluid Phase Model, Artificial Phosphatidylcholine Lipid Bilayers

Lipid	DPPC	DMPC	DLPC	DOPC	EPC
References	7,34	6	6	7	7
Temperature (°C)	50	30	30	30	30
Fatty acid structure	16:0	14:0	12:0	18:1	
Area per lipid molecule (A) (Ǻ^2^)	64	60.6	63.2	72.5	69.4
Radius per lipid molecule (Ǻ)		4.4		4.8	
Area per lipid headgroup (Ǻ^2^)	57				
Number of H2O molecules mixed with the lipid headgroup (n’_w_)	8.6	7.2	7.9	11.1	10.2

DPPC: dipalmitoyl-phosphatidylcholine; DMPC: dimyristoyl-phosphatidylcholine; DLPC: dilauroyl-phosphatidylcholine; DOPC: dioleoyl-phosphatidylcholine; EPC: egg phosphatidylcholine (a mixture of saturated and (poly)unsaturated PCs).

## LIST OF ABBREVIATIONS

DLPC: = dilauroyl-phosphatidylcholine
DMPC: = dimyristoyl-phosphatidylcholine
DOPC: = dioleoyl-phosphatidylcholine
DPPC: = dipalmitoyl-phosphatidylcholine
PEPC: = 1-palmitoyl-2-elaidoylphosphatidylcholine
POPC: = 1-palmitoyl-2-oleoylphosphatidylcholine
